# Examining the Impact of Permitless Firearm Legislation and COVID-19 on Crime and Arrests in Three Urban Cities

**DOI:** 10.1007/s11524-025-01024-4

**Published:** 2025-11-10

**Authors:** Nicholas Corsaro, Robin S. Engel, Jennifer M. Cherkauskas, Ryan T. Motz

**Affiliations:** 1 University of Cincinnati, Cincinnati, USA; 2 The Ohio State University, Columbus, USA

**Keywords:** Firearms legislation, COVID-19, Gun policy

## Abstract

While the number of state legislative changes to relaxed concealed firearm carrying laws continues to increase, research examining the impact of these laws on changes in criminal behavior, particularly in urban contexts, has not kept pace. To enhance our understanding of the potential impact of permitless carry legislative changes, we examined the temporal association between legislative changes and changes in illegal and dangerous behavior most likely to be associated with firearms violence and arrests in Lexington (KY), Oklahoma City (OK), and Tulsa (OK). We use statistical controls to account for a major temporal confounder: the disruption of social order that occurred during and after the global COVID-19 pandemic in 2020. Our findings show violent criminal offenses did not shift in the post-permitless carry period. However, there were consistent and robust statistically significant increases in illegal possession of a firearm, as well as an upward shift in threatening firearm behavior (i.e., brandishing a gun/pointing a firearm), net of controls and confounders. We also find significant and sizeable increases in stolen and recovered firearms in Lexington (KY), the lone setting that collected this outcome measure during our study period. We conclude by discussing how the findings can inform policy, forthcoming legislative initiatives, and future research.

## Introduction

Permitless carry legislation, which allows individuals to carry a handgun (openly or concealed) without a permit, is growing in popularity and has several variations. Twenty-four states enacted permitless carry legislation between 2014 and 2024, indicating permitless carry is an emerging legislative priority, given that 27 of the 50 states have passed such laws [1]. Importantly, scholarly research has explored the impact of several types of state firearms laws, including universal background checks, “may issue” (where there is discretion to withhold a permit even if a subject meets statutory requirements), “shall issue” (where a permit is granted if all legal requirements are met), and permitless carry [2–4]. The critical difference between permitless carry and other firearm carrying legislation is the elimination of the once-required background checks, proficiency testing, or training requirements [5].

Collectively, research findings have not demonstrated a robust pattern regarding the impact of firearm carrying legislation on reported violence. Such inconsistencies may be attributed to several factors, including variations across: (1) state legislative requirements (e.g., training criteria, disqualifying factors) [6, 7], (2) time periods of inquiry, (3) geographic contexts (e.g., state, county, and city-level studies), (4) research methodologies, and (5) analytical techniques [8]. A recent comprehensive review of studies examining the effects of firearm legislation on a range of outcomes concluded there was some evidence of increases in homicides (total and firearm homicides) and violent crime, moderate evidence of increases in felonious assaults, and limited evidence of increases in robberies [9]. However, these conclusions are about the impact of “shall-issue” concealed carry laws; fewer studies have examined the effects of permitless carry laws.

Permitless carry laws are considered a form of relaxed “right to carry” laws. We therefore expect permitless carry laws to have a potential association with crime and arrest outcomes of interest [10]. However, rigorous research evaluating permitless carry law’s impact is sparse and inconclusive [11]. A recent literature review identified a few studies specifically examining the effects of permitless carry laws and found inconclusive evidence for their impact on total homicides, firearm homicides, violent crimes, total and firearm suicides, and firearms purchases [9]. There is limited evidence showing permitless concealed carry laws coincide with increases in officer-involved shootings [12]. However, few studies have examined outcomes beyond homicide and serious violence, making it imperative that changes in firearm-carrying behavior, illicit activities, and community risk continue to be explored [12].

We present findings from research that examine the association between permitless carry legislation and serious violent crime, as well as threatening, aggressive, and reckless firearm behavior, such as brandishing firearms menacingly, illegal discharges of firearms, and lost or stolen firearms. We explore such potential impacts in three urban areas in Kentucky and Oklahoma, where permitless carry was enacted in 2019.

Four months later in Oklahoma and eight months later in Kentucky, the onset of the COVID-19 global pandemic in mid-March 2020 ushered in a period of in-home sheltering, attenuated social ties due to physical and social distancing, and weakened connections to social systems [13]. A large body of evidence has since shown that such a major disruption to people’s daily lives manifested heightened levels of aggression and antisocial behavior across various domains. Research in other fields, including those focusing on healthcare professionals [14,] school administrators [15], and airline industries [16], demonstrates increases in overall violence and aggression in the period following COVID-19. In addition, national concern sparked by the police killing of George Floyd in late May 2020 further contributed to social disruption.

In summary, we contend that less restrictive firearm laws that promoted increased weapon carrying during such a volatile period could have potentially served as a catalyst for threatening firearm-related behavior. Consequently, variation in firearm violence is a critical outcome of interest. However, we also hypothesize that changes in permitless carry legislation at the state level that preceded a period of civil upheaval (i.e., post-COVID-19 and immediately occurring social disturbances) are likely to be associated with increased incidences of risky, antisocial, threatening, and reckless firearm-related behavior in urban settings. Accordingly, our study includes statistical parameters for both the change in enacted firearm legislation and post-COVID-19 periods. The behaviors of interest are measured as: (1) firearm-related violent crimes, (2) arrests with various firearms-related charges, and (3) stolen/recovered firearms.

## Study Sites

In Kentucky, *Senate Bill 150* became effective in July 2019; it stated, “any person aged twenty-one or older, and otherwise able to lawfully possess a firearm, may carry concealed firearms or other concealed deadly weapons without a license” [17]. Likewise, *Oklahoma House Bill 2597* became effective in November 2019; it stated that “the carrying of a firearm, concealed or unconcealed, loaded or unloaded” shall not be prohibited for persons twenty-one years of age or older or by a person who is eighteen (18) years of age or older *and* a member or honorably discharged veteran of the United States Armed Forces, Reserves or National Guard, “and the person is otherwise not disqualified from the possession or purchase of a firearm under state or federal law and is not carrying the firearm in furtherance of a crime” [18].

The impact of these legislative changes on violence and other risky and threatening gun-related behaviors is examined in three densely populated urban areas within these states (Lexington, KY, Oklahoma City, OK, and Tulsa, OK), while simultaneously exploring the outcome changes post-COVID-19 (April 2020 onward).^1^ Based upon the 2020 US Census, Lexington is the second-largest city in Kentucky and 60^th^-largest city in the US, with a population of 322,570 [19]. The Lexington Police Department (LPD) had 602 sworn officers and 90 professional staff in 2019 [20]. Drawing upon violent crime data reported to the Federal Bureau of Investigation (FBI) in 2018 (the year preceding the change in permitless carry legislation in both states), Lexington accounted for 982 (~ 22%) of Kentucky's 3,454 reported violent crimes.

As the state capital and largest city in Oklahoma, Oklahoma City has a population of 687,725 as of the 2020 US Census [21], which ranks it as the 20th largest US city. In 2019, the Oklahoma City Police Department (OKCPD) had 1,173 sworn officers and 282 civilian employees [19]. The second largest city in Oklahoma is Tulsa, with 411,401 residents in the 2020 US Census [22], making it the 47th largest city in the US. Policing services are provided by the Tulsa Police Department (TPD), which comprised approximately 842 sworn officers and 195 civilian employees in 2019 [19]. Combined, Oklahoma City and Tulsa accounted for 61.9% (9,957/16,064) of all violent crimes reported in Oklahoma [20]. Collectively, these three cities were among the highest-risk urban locations for violence (including firearms violence) in Kentucky and Oklahoma.^2^

## Data and Analyses

Seven years of data (Jan 1, 2016 – Dec 31, 2022) were gathered from each study site. A total of 84 monthly observations were made for each outcome across the three cities. Lexington had 42 pre-intervention observations (Jan 1, 2016 – Jun 30, 2019) and 42 post-intervention observations (Jul 1, 2019 – Dec 31, 2022). For Oklahoma City and Tulsa, 46 pre-intervention observations (Jan 1, 2016 – Oct 31, 2019) and 38 post-intervention observations (Nov 1, 2019 – Dec 31, 2022) were included.

We obtained criminal offense, arrest, and stolen firearm data from criminal incident reports, arrest reports, and specialized investigation (motor vehicle theft) reports collected by the LPD, OKCPD, and TPD. The data included details of the criminal incidents (e.g., event dates and incident locations to ensure they occurred within city limits via ArcGIS boundary shapefiles) and arrests (e.g., date, location, and specific arrest charges). The unit of analysis is the incident level (reported crimes) or person (in-custody arrestee). For criminal offenses, standard Part I (serious) Uniform Crime Reports (UCR) violent crime data were available from LPD and TPD for this period. The OKCPD, however, changed its data collection and reporting system during the study period, impacting the reliability of the collection of aggravated assault and robbery incidents. Therefore, among reported violent outcome measures, only analyses of homicides are included for Oklahoma City.^3^

We also relied upon seven years of data documenting in-custody arrests and the associated criminal charges. Arrest data across the three agencies is operationalized as charge counts (monthly) by criminal charge type. We also assess total firearm arrests (arrests involving at least one firearm or gun-related offense charge) and arrests where at least one of the eligible/available charges was a “felon in possession.” The two Oklahoma departments also had arrest charges of “pointing of a firearm” and “illegal discharge of a firearm”; however, Lexington did not have information on these specific charges. Conversely, the LPD tracked incidents involving stolen and recovered firearms throughout the study period, while OKCPD and TPD did not readily capture this information.

### Interrupted Time Series Analyses

Ultimately, we utilized Generalized Linear Modeling (GLM) regression analysis (Long 1997) to estimate the impact of the statewide legislative changes regarding permitless carry on two categories of outcomes of interest: 1) violent criminal offenses (i.e., homicide, aggravated assaults, and robberies), and 2) charges within criminal arrests (i.e., firearm-related charges, illegal discharges of firearms, and illegal pointing of firearm arrest charges). Traditional linear (i.e., least squares) regression models are inappropriate for analyzing count outcomes because count data do not follow, or approximate, a normal distribution and thus analysis from these models would lead to biased and inconsistent estimates (King 1988). Therefore, each crime or arrest outcome modeled was estimated using a conditional Poisson distribution.^4^ The Poisson distribution can be written as follows:

$$P\left({Y}_{i}=\left.{y}_{i}\right|{X}_{i}\right)=\frac{\text{exp}(-\uplambda ){\uplambda }^{{y}_{i}}}{{y}_{i}!}$$

where *Y*_*i*_ is the random variable representing a count, *y*_*i*_ is a particular count value that denotes the number of monthly events (described above) that were observed for a given period (i.e., between January 2016 and December 2022), and where *λ*_*i*_ can take on different values for different cases. Given that our interest was to model the systematic variation observed for *λ*_*i*_ for each outcome, we relied upon the loglinear model:

$${\text{ln}\left({\lambda }_{i}\right)=\text{x}}_{i}^{T}\beta$$

where ${\text{x}}_{i}^{T}$ β is a linear combination of predictors for each case *i*, which in the current framework is as follows:

Intercept(β_0_) +Post-permitless carry dummy variable (β_1_) + post-COVID-19 dummy variable (β_2_) + monthly dummy variables (βx11 months (12 months – 1 month, the reference category)) + Error.^5^

For any regression model that showed evidence of a non-stochastic process (i.e., a statistically significant trend over time), we included (where needed) a third estimate, a beta coefficient in each such model, which was a linear-trend (i.e., sequential) variable (β_3_). As a sensitivity test, we estimated each final regression by outcome presented herein with an autoregressive term (using the “arpois” package in Stata), with none of the results indicating statistically significant autocorrelation in the residuals once we controlled for seasonal shocks via the dummy variables included in each regression (and linear trend, where needed).

Using monthly event counts, the “change” dates were operationalized centering on 1) the enacted statewide permitless carry legislation (in KY and OK) and 2) the April 2020 COVID-19 stay-at-home mandates and subsequent civil disruption via the follow-up period through December 2022.^6^ We relied upon a quasi-experimental interrupted time series methodology, frequently used to assess interventions’ distinct and unique impact, including legislative changes, on the association with behavior reported to and by police agencies. This technique has also proven helpful in examining the effects of the COVID-19 pandemic on changes in arrest rates, deviations in gun store sales practices that impacted criminals’ acquisition of new firearms, and the association between firearm laws and homicide rates in urban settings [8, 23, 24].

Time series analyses capture changes in the level and the slope in a longitudinal trend series after an intervention has been applied by comparing the parameters, shocks, and structures of the temporal dynamics to the pre-intervention periods. Consistent with prior research, we used fixed effects Poisson regression models on event counts for each study setting and outcome of interest. We also included monthly dummy variables to account for seasonality (excluded from the tables for ease of translation) and a COVID-19 indicator variable to control for the shift in the routine activities and social disruption associated with arrests and criminal offenses that corresponded with the April 2020 global pandemic (and the broad change in routine activities that it was associated with) [25]. All regression models controlled for time-varying factors and trends through the inclusion of monthly fixed effects.

## Results

None of the three urban study settings experienced a statistically significant shift in homicides after permitless carry legislation was enacted, overall or in the post-COVID-19 period. There were no statistically significant shifts in assaults or robberies in either the post-permitless carry or post-COVID-19 periods. Thus, the results in Table 1 demonstrate that periods after permitless carry and COVID-19 (i.e., period of social disruption) had no significant shifts in any of the reported Part I violent offenses. In addition, the estimated Incident Rate Ratios all corresponded with small effect sizes, indicating that even if the estimates had been statistically significant, the levels of violence were steady and consistent in each of these three study settings.

**Table 1:** Interrupted time series analyses for part I violent criminal offense distributions across study settings (1/2016–12/2022) – estimated change in offense levels, confidence intervals, and *p*-values

	β	St. Error	*p-value*	95% CI LL: UL	IRR (95% CI LL:UL)
*Homicide (All Sites)*					
Lexington					
Intercept (β_0_)	0.98	0.242	< 0.01	–-	–-
Post-Permitless Carry (β_1_)	0.115	0.201	0.567	−0.279: 0.509	1.12 (0.76: 1.66)
Post-COVID-19 (β_2_)	0.206	0.195	0.282	−0.170: 0.584	1.23 (0.84: 1.80)
Oklahoma City					
Intercept (β_0_)	1.71	0.217	< 0.01	–-	–-
Post-Permitless Carry (β_1_)	−0.201	0.146	0.167	−0.487: 0.084	0.81 (0.61: 1.08)
Post-COVID-19 (β_2_)	0.211	0.143	0.140	−0.069: 0.493	1.23 (0.93: 1.63)
Tulsa					
Intercept (β_0_)	1.47	0.228	< 0.01	–-	–-
Post-Permitless Carry (β_1_)	−0.118	0.145	0.413	−0.403: 0.165	0.88 (0.66: 1.18)
Post-COVID-19 (β_2_)	0.095	0.499	0.499	−0.180: 0.369	1.09 (0.84: 1.44)
*Aggravated Assault (Where Available)*					
Lexington					
Intercept (β_0_)	5.02	0.039	< 0.01	–-	–-
Post-Permitless Carry (β_1_)	−0.002	0.030	0.941	−0.061: 0.057	0.99 (0:94: 1.05)
Post-COVID-19 (β_2_)	0.045	0.028	0.115	−0.010: 0.101	1.04 (0.98: 1.10)
Tulsa					
Intercept (β_0_)	217.17	7.86	< 0.01	–-	–-
Post-Permitless Carry (β_1_)	0.023	0.025	0.365	−0.026: 0.073	1.02 (0.97: 1.08)
Post-COVID-19 (β_2_)	−0.014	0.035	0.687	−0.082: 0.054	0.98 (0.92: 1.05)
*Robbery (Where Available)*					
Lexington					
Intercept (β_0_)	3.88	0.086	< 0.01	–-	–-
Post-Permitless Carry (β_1_)	−0.012	0.088	0.890	−0.185: 0.160	0.98 (0.83: 1.17)
Post-COVID-19 (β_2_)	−0.009	0.079	0.900	−0.165: 0.145	0.99 (0.85: 1.15)
Linear trend (β_3_)	−0.013	0.002	< 0.01	−0.017: −0.008	0.98 (0.98: 0.99)
Tulsa					
Intercept (β_0_)					
Post-Permitless Carry (β_1_)	0.061	0.061	0.322	−0.059: 0.181	1.06 (0.94: 1.19)
Post-COVID-19 (β_2_)	0.029	0.069	0.673	−0.057; 0.206	1.07 (0.94: 1.23)
Linear trend (β_3_)	−0.012	0.001	< 0.01	−0.016: −0.009	0.98 (0.98: 0.99)

IRR = Incident Rate Ratios (IRRs)

Monthly dummy variables to control for seasonal shocks were included in each regression but are not displayed for ease of review

Table 2 provides the regression results for changes in types of firearm-related criminal charges within arrests across each urban setting. As we will document next, three sets of findings emerge from the various analyses: 1) the impact of the constitutional carry estimate was primarily associated with either no change or significant increases in overall firearm arrests, and in particular illegal possession of firearm arrests. There was little to no evidence of a statistically or substantively significant impact of constitutional carry on illicit behavior with a firearm (beyond illegal possession); 2) the post-COVID-19 impact was consistently observed with a significant increase in reckless and illicit gun behavior (such as illegal discharges and pointing of firearm charges) in the post-constitutional carry period across multiple study settings; 3) there was a statistically significant association between the post-constitutional carry estimate and stolen and recovered firearms in the lone study setting (Lexington) where the data were available – indicating that future studies should explore this association where such measures are collected by law enforcement.

**Table 2:** Interrupted time series analyses for criminal arrests by charge type and stolen and recovered firearm reports across study settings – estimated change in arrest levels, confidence intervals and *p*-values (1/2016–12/2022)

	β	St. Error	*p-value*	95% CI LL: UL	IRR (95% CI LL:UL)
**Broad Firearm Charges – All Sites**					
*Total Firearm Charges in Arrests*					
Lexington					
Intercept (β_0_)	2.69	0.169	< 0.01	–-	–-
Post-Permitless Carry (β_1_)	−0.041	0.104	0.690	−0.245: 0.162	0.95 (0.78: 1.17)
Post-COVID-19 (β_2_)	−0.097	0.102	0.341	−0.297: 0.103	0.91 (0.74: 1.10)
Oklahoma City					
Intercept (β_0_)	4.22	0.036	< 0.01	–-	–-
Post-Permitless Carry (β_1_)	0.194	0.056	< 0.01	0.083: 0.304	1.21 (1.08: 1.36)
Post-COVID-19 (β_2_)	0.148	0.062	0.017	0.026: 0.271	1.02 (1.02: 1.31)
Tulsa					
Intercept (β_0_)	1.47	0.228	< 0.01	–-	–-
Post-Permitless Carry (β_1_)	−0.118	0.145	0.413	−0.403: 0.165	0.88 (0.66: 1.18)
Post-COVID-19 (β_2_)	0.095	0.499	0.499	−0.180: 0.369	1.09 (0.84: 1.44)
*Illegal Firearm Possession Charges in Arrests*					
Lexington					
Intercept (β_0_)	5.02	0.039	< 0.01	–-	–-
Post-Permitless Carry (β_1_)	−0.002	0.030	0.941	−0.061: 0.057	0.99 (0:94: 1.05)
Post-COVID-19 (β_2_)	0.045	0.028	0.115	−0.010: 0.101	1.04 (0.98: 1.10)
Oklahoma City					
Intercept (β_0_)	3.68	0.067	< 0.01	–-	–-
Post-Permitless Carry (β_1_)	0.229	0.066	0.001	0.098: 0.360	1.26 (1.10: 1.43)
Post-COVID-19 (β_2_)	0.141	0.070	0.045	0.003: 0.280	1.15 (1.00: 1.32)
Tulsa					
Intercept (β_0_)	2.79	0.090	< 0.01	–-	–-
Post-Permitless Carry (β_1_)	0.155	0.090	0.076	−0.016: 0.325	1.16 (0.98: 1.38)
Post-COVID-19 (β_2_)	0.624	0.078	< 0.01	0.470: 0.778	1.86 (1.60: 2.17)
**Distinct Firearm Charges – Where Data Are Available**					
*Illegal Discharge of Firearm Charges in Arrests*					
Oklahoma City					
Intercept (β_0_)	1.28	0.177	< 0.01	–-	–-
Post-Permitless Carry (β_1_)	0.068	0.166	0.682	−0.258: 0.394	1.07 (0.77: 1.48)
Post-COVID-19 (β_2_)	0.495	0.161	< 0.01	0.179: 0.812	1.64 (1.19: 2.25)
Tulsa					
Intercept (β_0_)	0.43	0.229	0.057	–-	–-
Post-Permitless Carry (β_1_)	0.172	0.223	0.441	−0.266: 0.610	1.18 (0.76: 1.84)
Post-COVID-19 (β_2_)	0.484	0.218	0.026	0.057; 0.911	1.62 (1.05: 2.48)
*Pointing Firearm Charges in Arrests*					
Oklahoma City					
Intercept (β_0_)	1.47	0.146	< 0.01	–-	–-
Post-Permitless Carry (β_1_)	0.165	0.141	0.242	−0.111: 0.442	1.17 (0.89: 1.55)
Post-COVID-19 (β_2_)	0.339	0.141	0.017	0.059: 0.620	1.40 (1.06: 1.85)
Tulsa					
Intercept (β_0_)	1.72	0.242	< 0.01	–-	–-
Post-Permitless Carry (β_1_)	0.356	0.115	< 0.01	0.130: 0.582	1.42 (1.14: 1.79)
Post-COVID-19 (β_2_)	0.455	0.114	< 0.01	0.229: 0.681	1.57 (1.25: 1.97)
**Stolen and Recovered Firearms**					
Lexington					
Intercept (β_0_)	0.819	0.292	0.005	–-	–-
Post-Permitless Carry (β_1_)	0.379	0.226	0.094	−0.065: 0.823	1.46 (0.94: 2.27)
Post-COVID-19 (β_2_)	−0.721	0.260	0.006	−1.23: −0.211	0.48 (0.29: 0.80)
Linear trend (β_3_)	0.024	0.007	0.001	0.010: 0.038	1.04 (1.01: 1.04)

IRR = Incident Rate Ratios (IRRs)

Monthly dummy variables to control for seasonal shocks were included in each regression but are not displayed for ease of review

Regarding arrests with any firearm-related charge (i.e., illegal firearm possession, illegal discharge of a firearm, pointing of a firearm, etc.), within Oklahoma City we observed a statistically significant (*p* < 0.05) increase of roughly 21.4% (IRR = 1.21), which is a moderate effect size in firearm-related charges after permitless carry was enacted. Additionally, and above and beyond the impact of the post-legislative period, arrests with any firearm charges significantly (*p* < 0.05) increased by roughly 2.6% (IRR = 1.02) after COVID-19, which was a very small effect size. In sum, at least in Oklahoma City, there was a noteworthy increase in firearm-related charges within arrests. No such impacts were observed in Lexington or Tulsa.

Also displayed in Table 2, illegal possession of a firearm arrest charges increased in both Oklahoma City and Tulsa (with estimates that produced small to moderate effect sizes). Oklahoma City experienced a statistically significant increase in illegal firearm possession charges by roughly 25.7% (IRR = 1.257, *p* < 0.05) in the post-permitless carry period, above and beyond model controls. Similarly, illegal firearm possession arrest charges had a moderate statistically significant increase (16.7%) in Tulsa after permitless carry was enacted, though the effect size was smaller in magnitude (IRR = 1.16, *p* = 0.07). A post-COVID-19 increase in illegal firearm possession counts was observed in Tulsa, where charges increased by roughly 86.6% (IRR = 1.86, *p* < 0.01).

Next, Table 2 displays the results for distinct firearm charges, including illegal discharges of firearms and pointing firearms charges. In Oklahoma City, illegal discharges of firearms experienced a statistically significant increase of roughly 64.0% (IRR = 1.64, *p* = < 0.01) in the post-COVID period. Likewise, in Tulsa, illegal discharges of firearm charges increased by roughly 62.2% in the post-COVID period (IRR = 1.62, *p* = 0.026). Similar findings were observed for both Oklahoma cities when we examined changes in pointing of firearm charges, with a statistically significant increase in Oklahoma City by roughly 40.4% (IRR = 1.40, *p* = 0.017) and 57.6% in Tulsa (IRR = 1.57, *p* < 0.01) post-COVID. Thus, both sets of findings suggested a statistically significant increase with large effect sizes of reckless, dangerous, and illicit gun behavior post-COVID in both Oklahoma cities; similar outcomes were not available for analysis within Lexington.

Finally, Lexington was the lone urban setting that collected data on stolen and recovered firearms during the study. Lexington experienced a 46.1% increase in stolen and recovered guns (IRR = 1.46, *p* < 0.01), above and beyond a linear trend increase over time, and net of the reduction in the post-COVID period (likely to be related to a reduction in overall property crimes that occurred simultaneously during this period). It became readily apparent that the timing of the change in permitless carry in Lexington coalesced with the reported number of firearms stolen and recovered by the LPD (which anecdotally led to their focus on the collection of this measure to begin with).

## Discussion

The empirical results from this study provide a robust and consistent narrative. First, permitless carry legislation, coupled with the COVID-19 global pandemic and the nearly simultaneous disruption to social order that the April 2020 indicator variable is designed to capture, *did not* correspond with any significant change in violent criminal offenses most likely to be firearm-related (homicides, assaults, and robberies) across Lexington, Oklahoma City, or Tulsa. Whatever consequences were associated with relaxed firearm carrying did not manifest into shifts in violent offending in either a positive or negative direction.

Second, regression parameters estimating the impact of the enactment of permitless carry legislation and the COVID-19 global pandemic were associated with significant and, at times, sizeable increases in recklessness and unlawful behavior with firearms in each urban study setting. However, the specific changes in recklessness, the magnitude of those effects, and the timing of the observed shifts varied over time and across study locations. More generally, we observed statistically significant increases in total gun-related charges within arrests, illegal possession of firearms, and arrests where suspects were charged with illegally pointing a firearm. The association appeared clearer for illegal possession charges associated with the change in permitless carry.^7^ However, the precise timing of the change and the magnitude of the shift in reckless, threatening, and menacing firearm behavior seemingly varied by outcome, at least across the three major urban settings studied here.

Finally, we observed significant increases in stolen and recovered firearm counts in Lexington after permitless carry legislation was enacted. This finding is consistent with gun-theft research, which shows that individuals who are at the highest risk of having their firearm stolen own more than five firearms, carry their firearms for protection, reside in southern US States (KY and OK are both captured as Southern states via the census), and store their guns unsafely [26]. It is certainly possible that civilians began carrying firearms for protection in public, becoming a target for theft due to less-than-stellar storage practices. Subsequent focus group interviews with patrol officers in all three agencies included descriptions of rising numbers of traffic stops with motorists who placed their unsecured handguns in cupholders, passenger seats, and on top of floor mats after permitless carry legislation was passed. The data needed to empirically examine the impact of these types of changes in behavior were only available in one of the three study sites, suggesting that more research is needed that explores alternative measures beyond reported crime.

These patterns in the data arose despite two key limitations. First, our counterfactual is limited. We do not have arrest data in control settings within similar cities that did not experience changes in permitless carry legislation. Criminal charge and arrest data are not as readily available as criminal offense data, and future research needs to examine criminal charges within arrests to unravel whether the findings observed herein occurred elsewhere. Future research should also disentangle whether these findings are unique to densely populated urban settings like those examined in Lexington, Oklahoma City, or Tulsa, or whether the impact of permitless carry firearm laws varies for urban, suburban, and rural areas as research on other types of firearm laws demonstrates [27].

Second, the timeline of this study was distinct due to the almost simultaneous shifts in the routine activities of human behavior (permitless carry, the COVID-19 pandemic, and post-Floyd social unrest) – which impacted changes in crime and criminal behavior both directly and indirectly [28]. It is possible that the empirical shift in reckless and threatening firearm-related behavior we observed was a broader indicator of a general change in incivility that emerged over this particular period. It is impossible to precisely disentangle whether the changes we observed were a product of the enactment of permitless carry legislation alone or a multiplicative manifestation of increased incivility combined with changes in firearm carrying. While we did not find any other major firearm legislative changes in KY or OK during this study period, it is noteworthy that gun sales increased considerably in the U.S. in March 2020, with an estimated 40% increase in background checks in that month alone [29]. The results are substantively similar in regression models where we estimate one effect without the other. Thus, we estimated both parameters simultaneously to demonstrate which effect was likely more pronounced on a given outcome in each setting. Given that our post-COVID variable retained its indicator variable value (i.e., value = 1) from April 2020 (i.e., onset) through December 2022 (end of the study period), it would be extremely utilitarian from a methodological standpoint to examine study settings that had divergent constitutional carry periods to parse out potential interactions between COVID and constitutional carry legislation. Future studies could enhance the scholarly literature by comparing the measures used here in settings that changed their permitless carry laws pre-COVID (i.e., before 2017/2018 to have a long enough follow-up period) and those that enacted them in the post-COVID only periods (e.g., 2022–2023 onward).

Despite these limitations and the cautious interpretation that we provide here, we conclude the following: The cities of Lexington, Oklahoma City, and Tulsa were subjected to a heightened risk of dangerous, disorderly, and threatening gun behavior when background checks and gun training requirements were relaxed (i.e., when permitless carry legislation passed), particularly in the post-COVID-19 period. However, this shift in observed arrests and criminal charges did not translate to increased gun violence measured as Part I serious criminal offenses. We contend that future studies should examine patterns and trends beyond shifts in violent criminal offenses when considering the impact of easing permitless carry laws. Changes to other dangerous and disorderly behaviors during interactions with police should also be considered and can be measured using specific charges associated with gun-related arrests.

## Data Availability

The data for this study are archived with the National Archive of Criminal Justice Data (NACJD), a part of the Inter-University Consortium for Political and Social Research (ICPSR) at the University of Michigan.

## Footnotes

1 These three study sites were selected because at the time this project received funding from the National Institute of Justice, these two states (KY and OK) were the latest (at that time) to adopt such legislation – and the largest urban law enforcement agencies with some of each states highest crime rates agreed to participate in the study.

2 The research team relied on data retrieved directly from the police departments for this study. A change in leadership in the Louisville Metro Police Department (LMPD) resulted in a decision to discontinue their participation in the larger NIJ-funded research study in the immediate aftermath of several high-profile critical incidents and police uses of force, to focus their resources on protest response, the Department of Justice pattern and practice investigation, and officer recruitment, retention, and wellness.

3 OKPD experienced a change in their offense reporting system beginning in Jan 2019 (from UCR to the National Incident Based Reporting System, NIBRS). As a result, the counts for multiple-offense incidents increased. Thus, we only examined criminal offenses that had (a) measurement consistency over time, and (b) were likely to be impacted by the change in constitutional carry legislation (i.e., violent offenses).

4 All Poisson regression results presented relied on the use of STATA 18.0 software.

5 The COVID estimate is direct/main effect only. We did not include an interaction term in the models presented here-in due to data limitations (i.e., lack of independence between the COVID and constitutional carry variables given the primarily overlapping periods).

6 The George Floyd murder occurred in May 2020 and the subsequent civil unrest from these incidents and other related events were nearly simultaneous with the April 2020 COVID-19 date. Thus, the COVID-19 parameter in the models invariably captures the simultaneity of the disruption of social order emulating from these dates of interest. However, the April 2020 onward COVID-19 estimate is the onset of such disruption and is handled as such in the models.

7 It is noteworthy that when we examined the various outcomes with only the post-COVID estimates and then only with the post-constitutional carry estimates (i.e., not controlling for the simultaneous impact of both estimates), the results are very similar with those presented here-in (where both sets of estimates are simultaneously included in the models). Given the significant overlap of these two time periods within this study, we strongly recommend (in our discussion of limitations) that future studies should examine settings that have some variation in these two time periods to assess which temporal impacts are most pronounced with which outcomes (given the stability observed in these findings).
